# Supplementary material for: The nucleic acid binding protein SFPQ represses EBV lytic reactivation by promoting histone H1 expression
Source: Nat Commun. 2024 May 16;15:4156. doi: 10.1038/s41467-024-48333-x (PMC11099029; doi:10.1038/s41467-024-48333-x)
Supplement: Supplementary file 1 — Supplementary Information [file 41467_2024_48333_MOESM1_ESM.pdf]

The Nucleic Acid Binding Protein SFPQ Represses EBV Lytic Reactivation by Promoting Histone H1 Expression

Murray-Nerger, et. al.

Supplementary Information

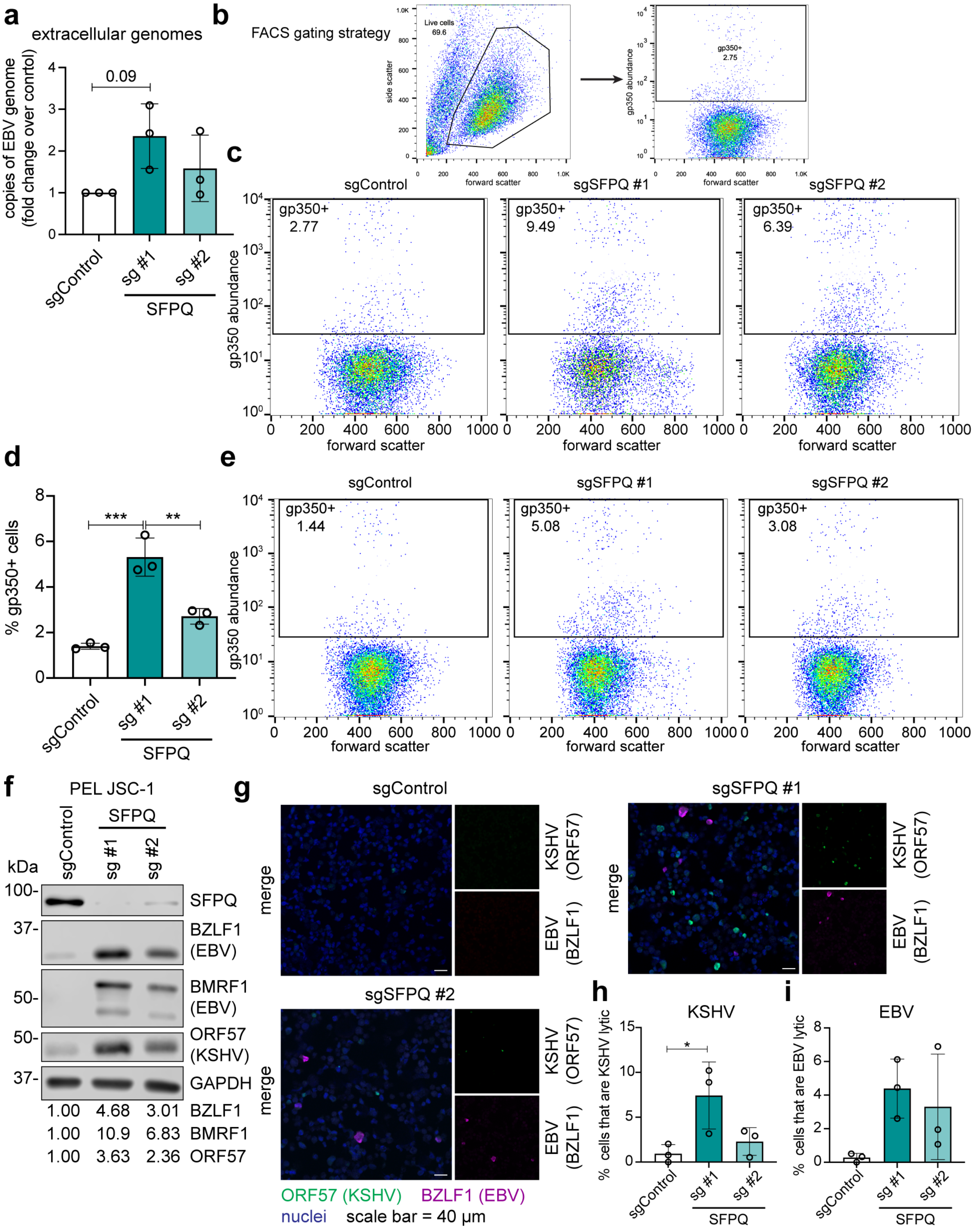

Supplementary Figure 1. SFPQ represses gammaherpesvirus lytic reactivation. a Mean ± standard deviation from n = 3 biological replicates of extracellular EBV genome copy number from Cas9+ P3HR-1 cells expressing control or SFPQ sgRNAs. *P*-value calculated by one-way ANOVA is shown. **b** FACS gating strategy for gp350 abundance quantification. **c**

Representative FACS plots of gp350 abundance in Cas9+ P3HR-1 cells following expression of control or SFPQ sgRNAs. **d** Mean ± standard deviation of % gp350+ cells from n = 3 biological replicates of Cas9+ MUTU I cells expressing control or SFPQ sgRNAs. **e** Representative FACS plots of gp350+ abundance in Cas9+ MUTU I cells expressing control or SFPQ sgRNAs. **f** Immunoblot representative of n = 3 biological replicates of the indicated EBV and KSHV proteins from whole cell lysate (WCL) obtained from Cas9+ PEL JSC-1 cells expressing control or SFPQ sgRNAs. Densitometry quantification with values normalized to the loading control GAPDH is shown. **g** Representative immunofluorescence images from n = 3 biological replicates of Cas9+ PEL JSC-1 cells expressing control or SFPQ sgRNAs. KSHV ORF57+ (green) or EBV BZLF1+ (magenta) cells are shown. Nuclei are labeled with DAPI (blue). Scale bar is 40 μm. **h-i** Quantification of **h** % KSHV ORF57+ or **i** % EBV BZLF1+ Cas9+ PEL JSC-1 cells following expression of control or SFPQ sgRNAs. Mean percentage ± standard deviation from n = 3 biological replicates is shown. * $P \leq 0.05$, ** $P \leq 0.01$, *** $P \leq 0.001$, calculated by one-way ANOVA. Source data are provided as a Source Data file.

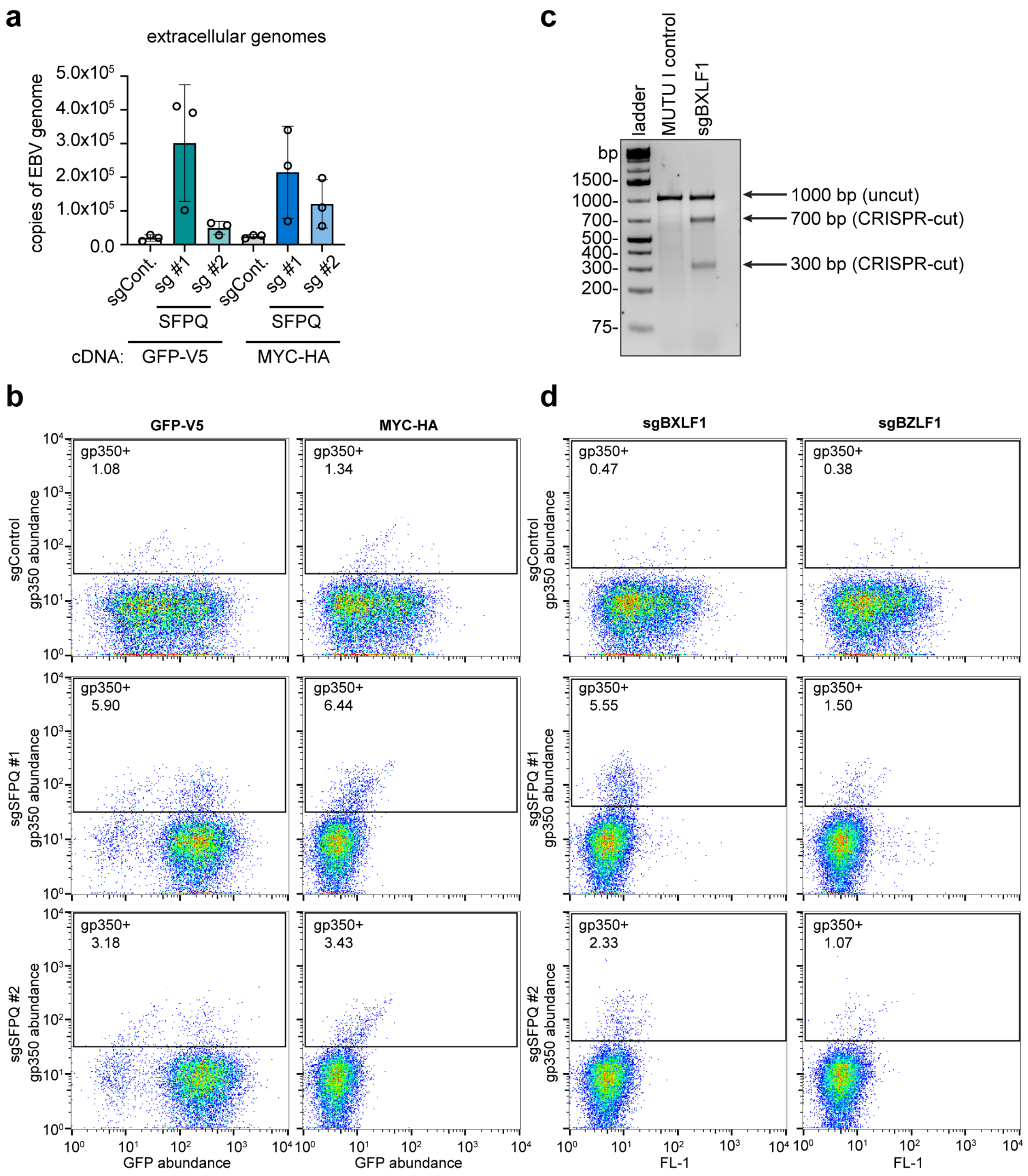

Supplementary Figure 2. SFPQ represses EBV lytic reactivation independently of MYC at the immediate early stage of lytic reactivation. a Mean ± standard deviation of number of extracellular EBV genome copies from n = 3 biological replicates of Cas9+ MUTU I cells expressing GFP-V5 or MYC-HA cDNA and also expressing control or SFPQ sgRNAs. **b** Representative FACS plots of gp350 abundance in Cas9+ MUTU I cells expressing GFP or MYC cDNA and control or SFPQ sgRNAs. **c** T7E1 ligase assay assessing the extent of Cas9 editing of the EBV genomic *BXLF1* locus. Briefly, a PCR around the *BXLF1* locus is performed in both control and sgBXLF1 expressing cells. The PCR products are hybridized and then

incubated with T7E1 ligase, which will cleave mis-matched DNA. Mis-matched DNA between the control and sgBXLF1 samples will occur when the *BXLF1* locus has been successfully cut by Cas9. The genome targeting efficiency can be assessed by comparing the summed ratio of the two lower bands (products of the T7E1-mediated cleavage) to the upper uncut band (no mis-matches and therefore not cleaved by T7E1). More detailed protocol information can be found in the methods. **d** Representative FACS plots of gp350 abundance in Cas9+ MUTU I cells following expression of BXLF1 or BZLF1 sgRNAs and also control or SFPQ sgRNAs. There is signal in the FL-1 channel in the sgControl samples because the PXPR construct, which expresses GFP, was used as the control sgRNA in this case.

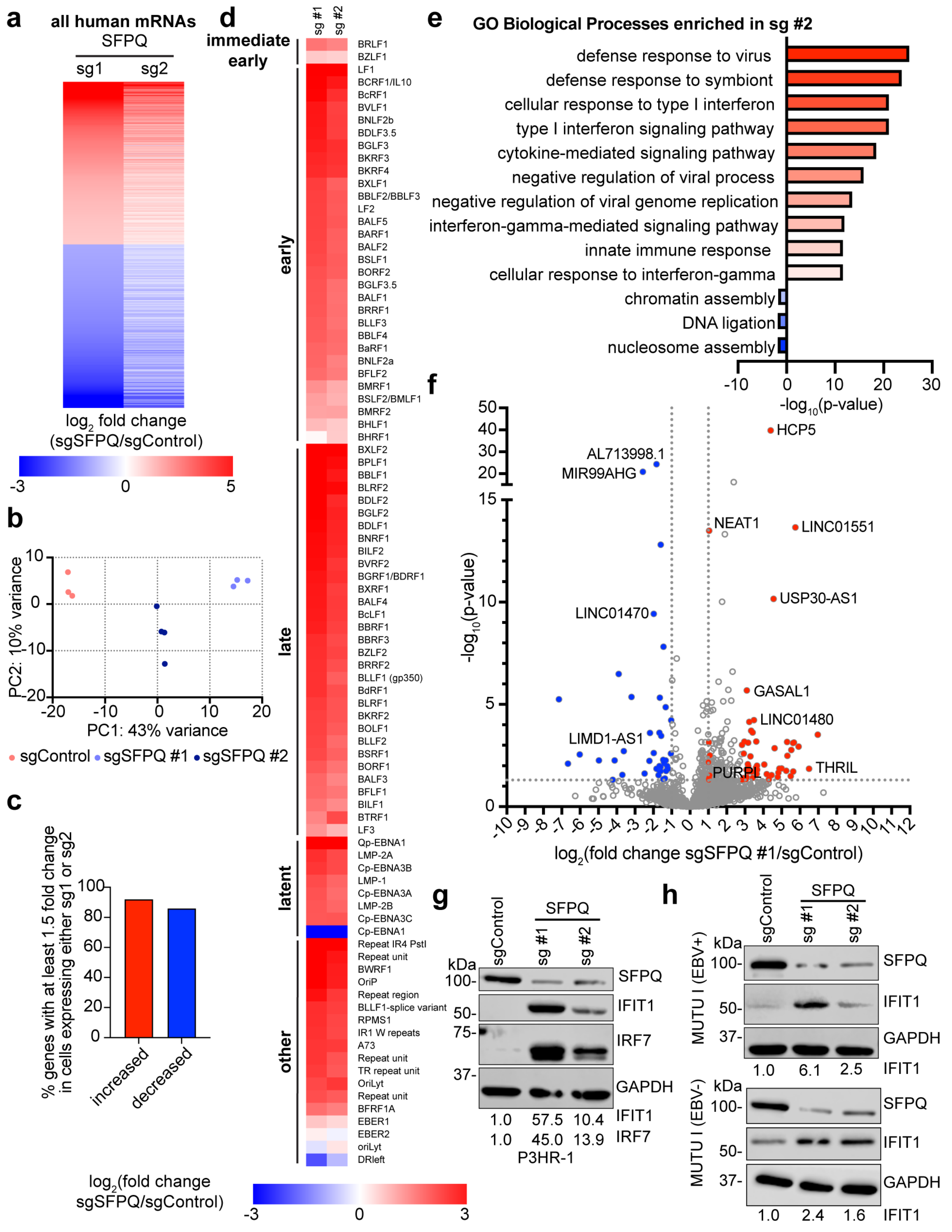

Supplementary Figure 3. SFPQ regulates gene expression. a Heatmap showing the similar log₂ fold change trends in host cell mRNA abundance observed upon expression of two

independent SFPQ sgRNAs relative to mRNA levels in cells with control sgRNA expression. **b** Principal component (PC) analysis plot comparing biological triplicate RNA-seq values from cells expressing control (pink), SFPQ #1 (light blue), or sgSFPQ #2 (dark blue) sgRNAs. Of note, replicate 2 of SFPQ sgRNA #2 was run in technical duplicate. **c** Percentage of genes that displayed at least a 1.5 fold increase or decrease in abundance upon expression of both of the SFPQ sgRNAs relative to expression in cells with control sgRNA expression. **d** Heatmap showing the similar $\log_2$ fold change trends in EBV-encoded mRNA abundances observed upon expression of SFPQ sgRNAs relative to mRNA levels in cells with control sgRNA expression. **e** Significantly enriched (adjusted p-value < 0.05) gene ontology (GO) Biological Processes that were increased or decreased in abundance in cells expressing SFPQ sgRNA #2 relative to levels in cells with control sgRNA expression. **f** Volcano plot of the $-\log_{10}$(p-value) vs. $\log_2$(fold change) of host cell lncRNA expression in cells expressing SFPQ sgRNA #1 vs. control sgRNA. Selected significantly changed lncRNAs are highlighted in red (increased) or blue (decreased). **g** Immunoblot of IFIT1 and IRF7 from WCL obtained from Cas9+ P3HR-1 cells following expression of control or SFPQ sgRNAs. **h** Immunoblots of IFIT1 from WCL obtained from Cas9+ EBV+ MUTU I or EBV- MUTU I cells following expression of control or SFPQ sgRNAs. Representative immunoblots from n = 3 biological replicates and densitometry quantification with values normalized to the loading control GAPDH are shown. Source data are provided as a Source Data file.

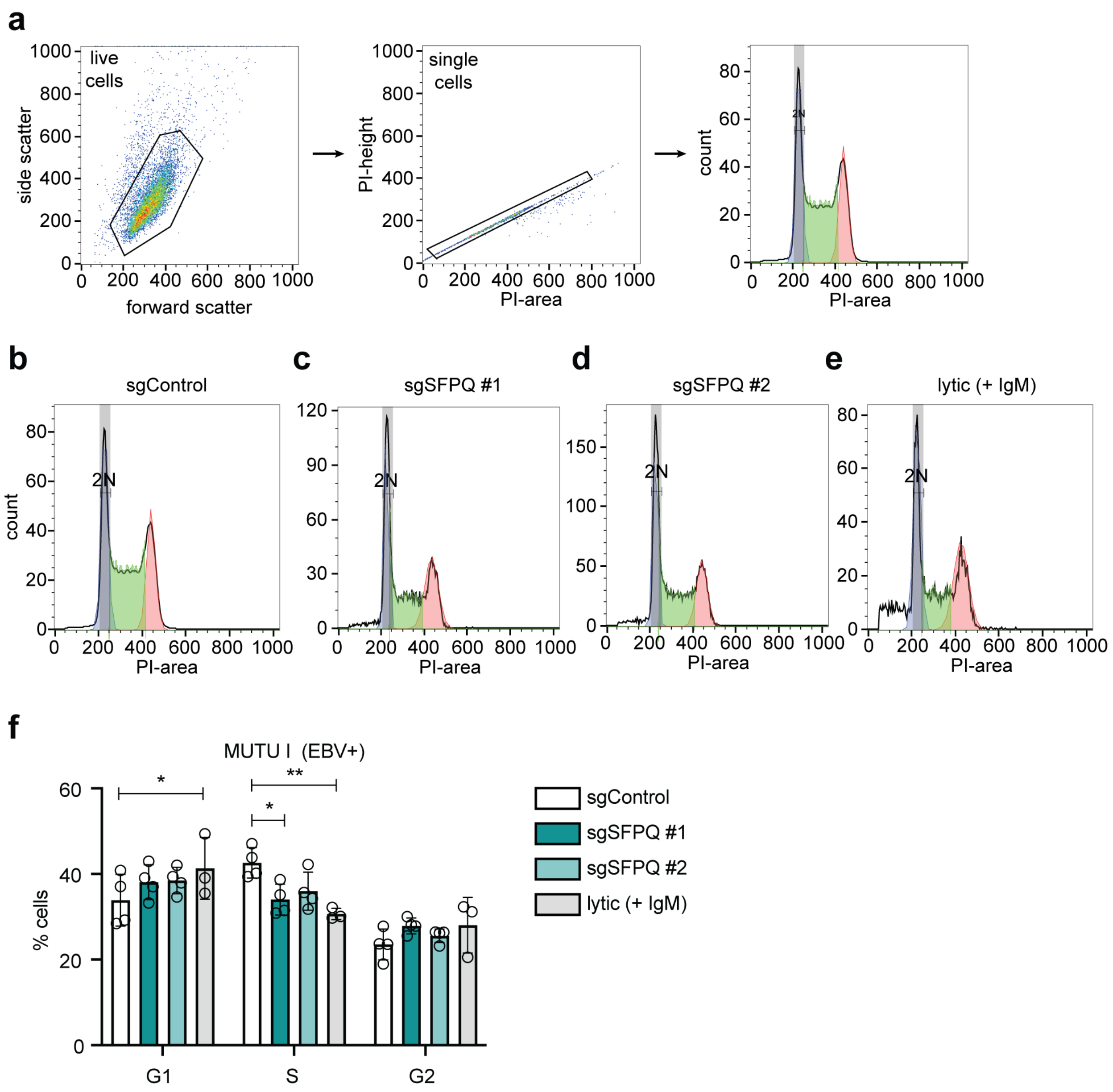

Supplementary Figure 4. SFPQ depletion does not alter cell cycle stage relative to α-IgM induced lytic reactivation. a FACS gating strategy for cell cycle quantification. **b-e** Representative Cas9+ MUTU I cell cycle propidium iodide (PI) analysis plots obtained at 6 days following expression of control or SFPQ targeting sgRNAs or upon lytic reactivation via α-IgM crosslinking for 24 h. **f** Mean ± standard deviation of % cells in each of the cell cycle phases (G1, S, G2) upon expression of either control or SFPQ targeting sgRNAs in Cas9+ MUTU I (EBV+) cells or upon α-IgM crosslinking from n = 3-4 biological replicates. * $P \leq 0.05$, ** $P \leq 0.01$, calculated by two-way ANOVA.

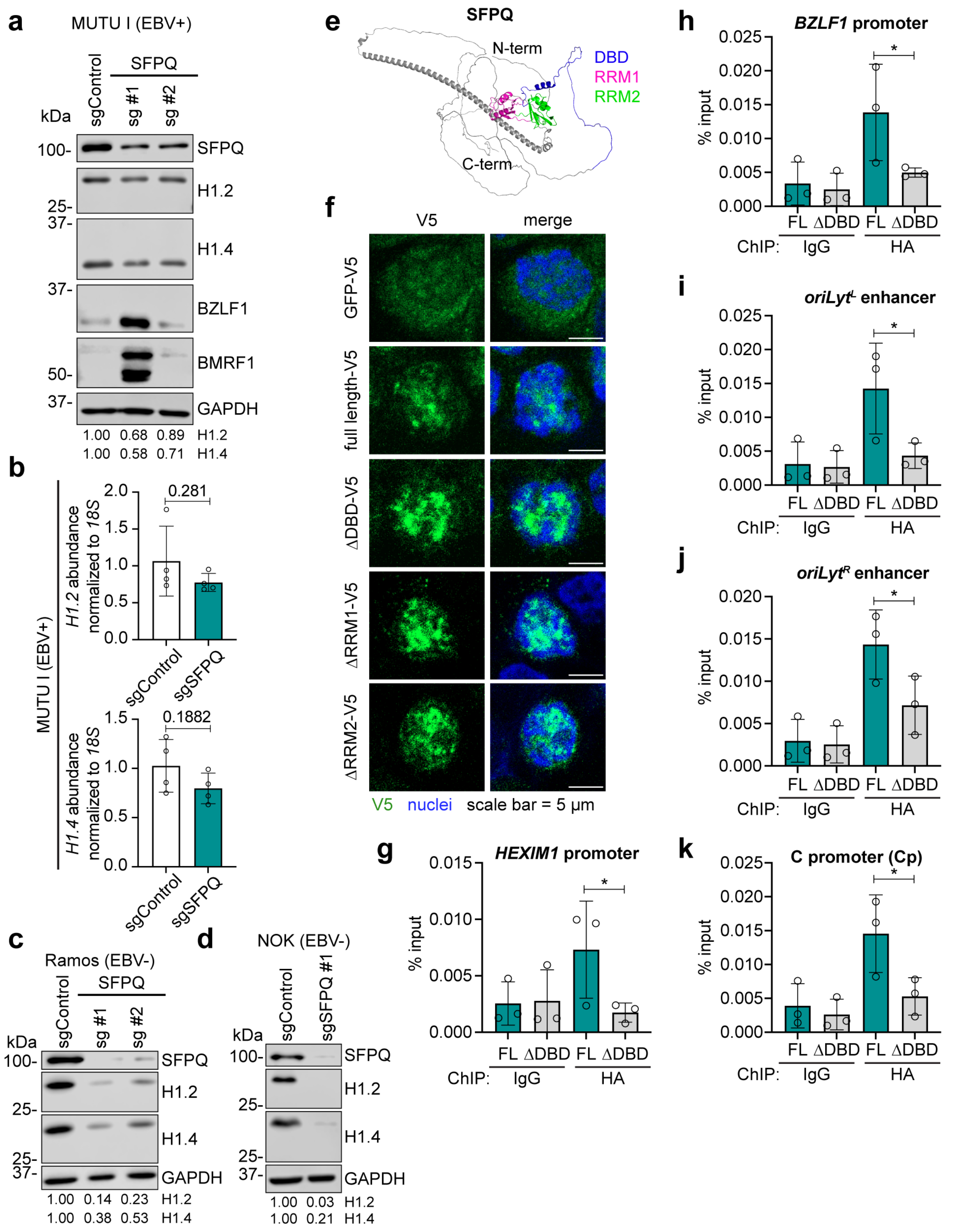

Supplementary Figure 5. SFPQ regulates expression of histone H1 variants. a Immunoblot of the indicated proteins from WCL obtained from Cas9+ EBV+ MUTU I cells following expression of control or SFPQ sgRNAs. **b** RT-qPCR analysis of mean ± standard deviation of

18S RNA normalized *HIST1H1C (H1.2)* and *HIST1H1E (H1.4)* abundances in Cas9+ EBV+ MUTU I cells following expression of control or SFPQ sgRNAs from n = 4 biological replicates. *P*-values calculated by a two-tailed Student's t-test are shown. **c** Immunoblot of the indicated proteins from WCL obtained from Cas9+ EBV- Ramos B cells expressing control or SFPQ sgRNAs. **d** Immunoblot of the indicated proteins from WCL obtained from Cas9+ normal oral keratinocytes (NOK) expressing control or SFPQ sgRNAs. **e** Alpha-fold prediction of SFPQ structure with key domains highlighted. **f** Representative immunofluorescence images of Cas9+ EBV+ MUTU I cells expressing control GFP-V5, SFPQ-V5 full length or SFPQ-V5 with domain deletions (ΔDBD, ΔRRM1, ΔRRM2) cDNAs (green) in EBV+ MUTU I cells. cDNA constructs are shown in green and nuclei (DAPI) are shown in blue. Scale bar = 5 μm. **g-k** ChIP-qPCR analysis of HA-FL, HA-ΔDBD, or control IgG occupancy at the **g** *HEXIM1* promoter, **h** *BZLF1* promoter, **i** $oriLyt^{L}$ enhancer, **j** $oriLyt^{R}$ enhancer, or **k** C promoter (Cp) in Cas9+ EBV+ MUTU I cells. Mean ± standard deviation from n = 3 biological replicates is shown. * $P \leq 0.05$ calculated by one-way ANOVA test. Representative immunoblots from n = 3 biological replicates and densitometry quantification with values normalized to the loading control GAPDH are shown. Source data are provided as a Source Data file.

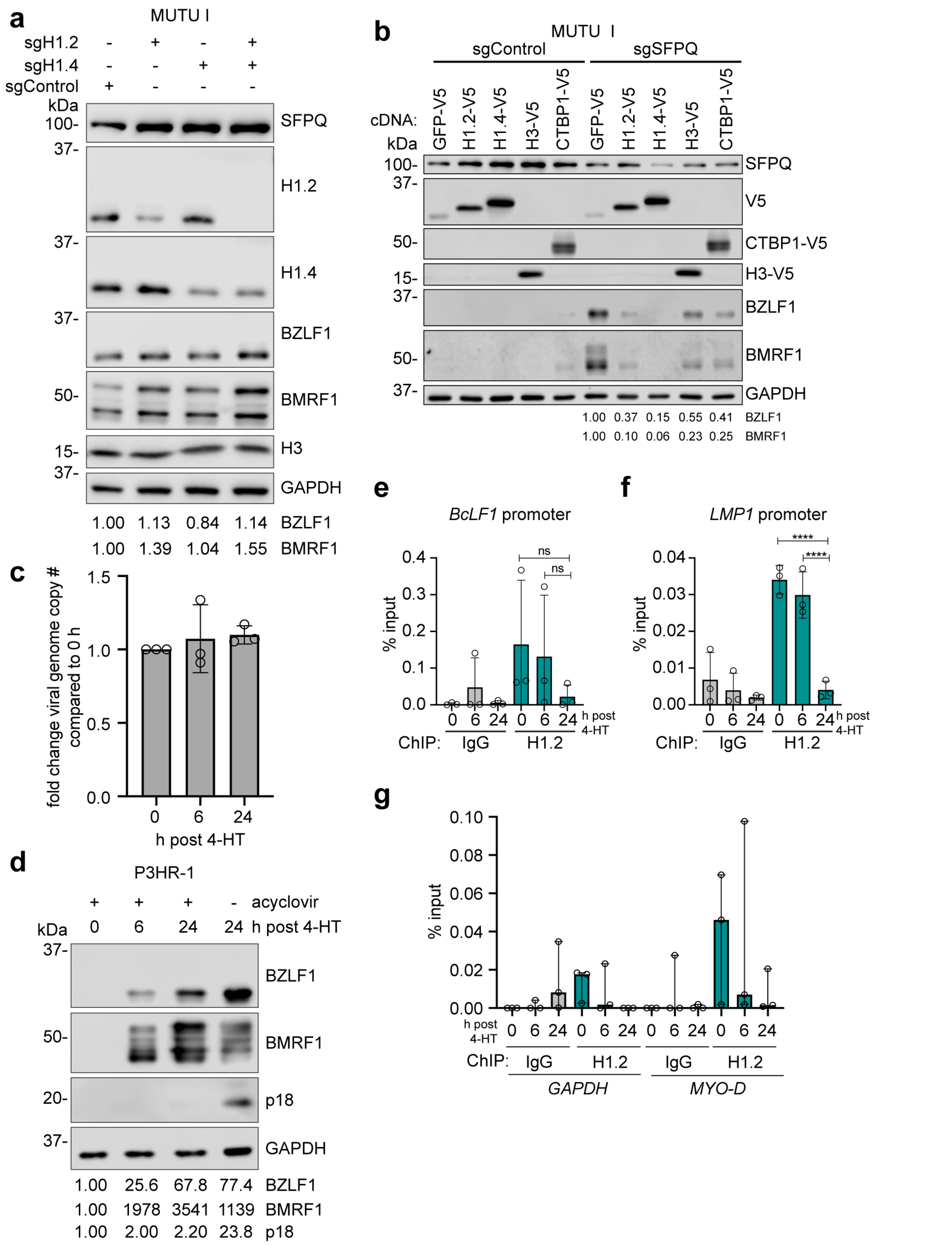

Supplementary Figure 6. H1.2 association with the EBV genome declines during early stages of lytic reactivation. a Immunoblot of WCL from Cas9+ EBV+ MUTU I cells that expressed control sgRNA or sgRNA targeting H1.2 and/or H1.4 for 9 days. **b** Immunoblot of

WCL from Cas9+ EBV+ MUTU I cells following expression of GFP, H1.2, H1.4, H3 or CTBP1 cDNAs as well as control or SFPQ sgRNAs, as indicated. **c** qPCR analysis of viral genome copy number from P3HR-1 ZHT/RHT cells triggered for lytic reactivation by 500 μM 4-HT in the presence of 100 µg/mL acyclovir for the indicated hours. Mean $\pm$ standard deviation from n = 3 biological replicates is shown. **d** Immunoblot analysis of WCL from Cas9+ P3HR-1 cells treated as in panel c with 4-HT for the indicated times in the absence or presence of acyclovir. **e-f** ChIP-qPCR analysis of H1.2 occupancy at the EBV genomic ***e*** *BcLF1* and **f** *LMP1* promoters at 0, 6, and 24 h post lytic reactivation via 4-HT treatment of Cas9+ P3HR-1 cells in the presence of acyclovir. Mean $\pm$ standard deviation from n = 3 biological replicates is shown. **** $P \leq 0.0001$, ns not significant calculated by one-way ANOVA. **g** ChIP-qPCR analysis of negative control IgG vs. H1.2 occupancy of the *GAPDH* and *MYO-D* promoters at 0, 6, and 24 h post lytic reactivation via 4-HT treatment of Cas9+ P3HR-1 cells in the presence of acyclovir. Median $\pm$ 95% confidence interval from n = 3 biological replicates is shown. Representative immunoblots from n = 3 biological replicates and densitometry quantification with values normalized to the loading control GAPDH are shown. Source data are provided as a Source Data file.

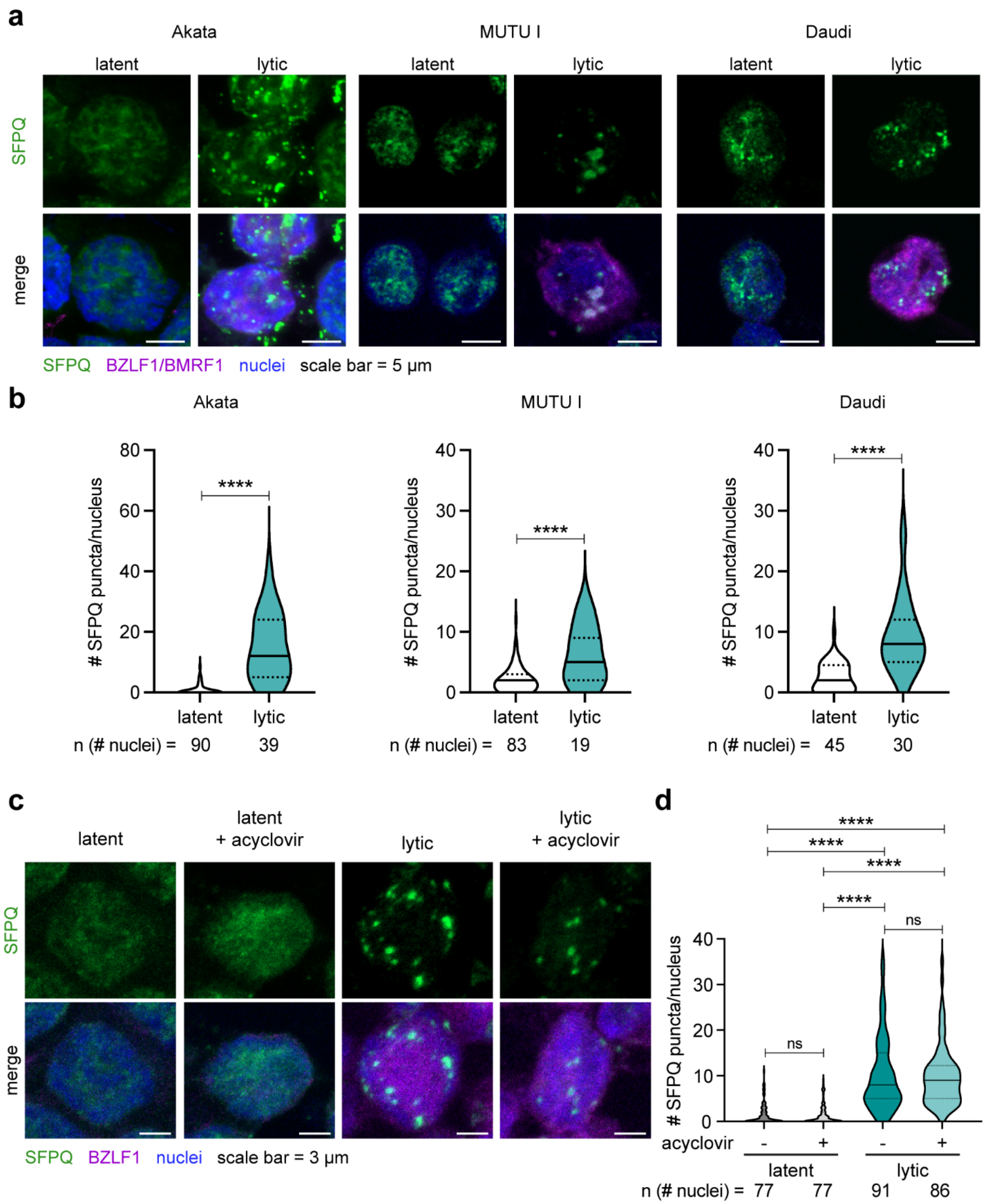

Supplementary Figure 7. SFPQ is re-distributed within the nucleus during early stages of EBV lytic reactivation. a-b a Representative images and **b** quantification of SFPQ re-distribution in latent and lytic (24 h post reactivation) Burkitt lymphoma cells. Data is shown for Akata (induced with αIgG), MUTU I (induced with αIgM), and Daudi (induced with sodium

butyrate and TPA) cells. Quantification of the number of SFPQ puncta per nucleus across each condition is shown. Violin plots show median (solid line) and interquartile range (dotted lines). The number of nuclei quantified across at least 2 biological replicates is indicated. **** $P \leq 0.0001$ calculated by two-tailed Mann-Whitney t-test. **c** Representative images of SFPQ localization in P3HR-1 ZHT/RHT cells +/- acyclovir in latency or 24 h post lytic induction by 4-HT. **d** Quantification of the number of SFPQ puncta per nucleus across the four conditions shown in panel c. Violin plot showing the median (solid line) and interquartile range (dotted lines) is shown. The number of nuclei quantified across n = 3 biological replicates are indicated. **** $P \leq 0.0001$, ns not significant calculated by one-way ANOVA. Source data are provided as a Source Data file.

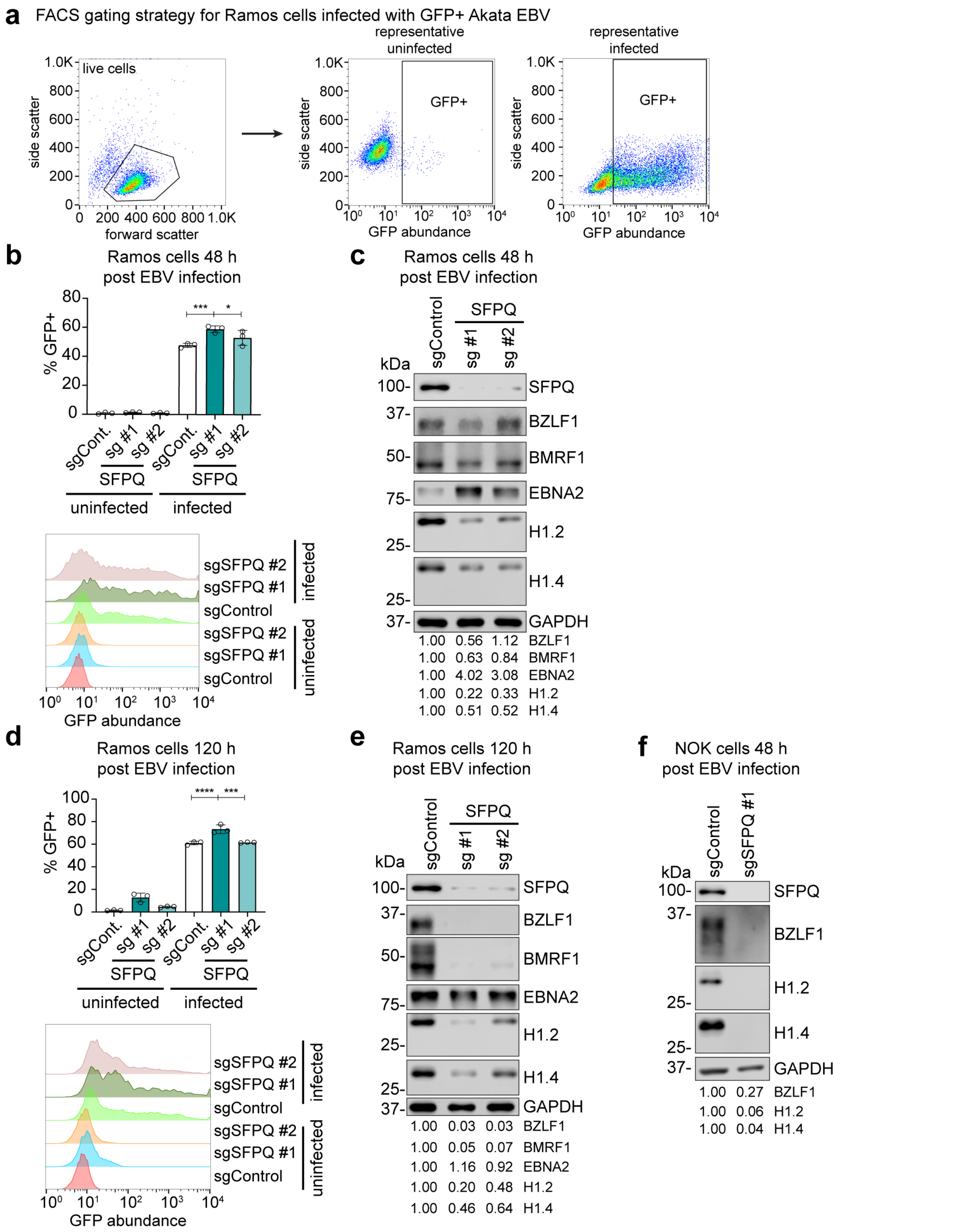

Supplementary Figure 8. SFPQ depletion impacts EBV encoded protein expression at early stages of infection. a FACS gating strategy for Ramos cells infected with GFP+ Akata

EBV. Representative dot plots for EBV- Ramos B-cells mock infected or infected with recombinant Akata EBV encoding a GFP transgene. The same gate with used for both conditions. **b** Mean ± standard deviation % GFP+ Ramos cells from n = 3 biological replicates of Cas9+ Ramos cells expressing control or SFPQ sgRNAs at 48 h post mock infection or infection with GFP+ Akata EBV (top). A representative FACS histogram is shown (bottom). **c** Immunoblot analysis of WCL from Cas9+ Ramos cells expressing control or SFPQ sgRNAs at 48 h post infection with GFP+ Akata EBV. **d** Mean ± standard deviation % GFP+ Ramos cells from n = 3 biological replicates of Cas9+ Ramos cells expressing control or SFPQ sgRNAs at 120 h post mock infection or infection with GFP+ Akata EBV (top). A representative FACS histogram is shown (bottom). **e** Immunoblot of WCL from Cas9+ Ramos cells expressing control or SFPQ sgRNAs at 120 h post infection with GFP+ Akata EBV. **f** Immunoblot of WCL from Cas9+ NOK cells expressing control or SFPQ sgRNAs at 48 h post infection with GFP+ Akata EBV. Representative immunoblots from n = 3 biological replicates and densitometry quantification with values normalized to the loading control GAPDH are shown. * $P \leq 0.05$, *** $P \leq 0.001$, ****, $P \leq 0.0001$ were calculated by one-way ANOVA. Source data are provided as a Source Data file.
