# Supplementary material for: The nucleic acid binding protein SFPQ represses EBV lytic reactivation by promoting histone H1 expression
Source: Nat Commun. 2024 May 16;15:4156. doi: 10.1038/s41467-024-48333-x (PMC11099029; doi:10.1038/s41467-024-48333-x)
Supplement: Supplementary file 3 — Description of Additional Supplementary Files [file 41467_2024_48333_MOESM3_ESM.pdf]

Description of Additional Supplementary Files

File Name: Supplementary Data 1
Description: RNA-seq data analysis comparing gene expression in Cas9+ P3HR-1 cells expressing sgSFPQ versus sgControl sgRNAs. Each tab shows the data analysis for the sgSFPQ guide of interest (sg #1 or sg #2) compared to the sgControl for either human or EBV genes.

File Name: Supplementary Data 2
Description: Primers used in this study.

File Name: Supplementary Data 3
Description: Antibodies used in this study.
