# Supplementary material for: The nucleic acid binding protein SFPQ represses EBV lytic reactivation by promoting histone H1 expression
Source: Nat Commun. 2024 May 16;15:4156. doi: 10.1038/s41467-024-48333-x (PMC11099029; doi:10.1038/s41467-024-48333-x)
Supplement: Supplementary file 7 — Reporting Summary [file 41467_2024_48333_MOESM7_ESM.pdf]

nature portfolio

Corresponding author(s): Benjamin Gewurz

Last updated by author(s): 4/16/2024

Reporting Summary

Nature Portfolio wishes to improve the reproducibility of the work that we publish. This form provides structure for consistency and transparency in reporting. For further information on Nature Portfolio policies, see our Editorial Policies and the Editorial Policy Checklist.

Statistics

For all statistical analyses, confirm that the following items are present in the figure legend, table legend, main text, or Methods section.

n/a	Confirmed	
☐	☒	The exact sample size (*n*) for each experimental group/condition, given as a discrete number and unit of measurement
☐	☒	A statement on whether measurements were taken from distinct samples or whether the same sample was measured repeatedly
☐	☒	The statistical test(s) used AND whether they are one- or two-sided<br>*Only common tests should be described solely by name; describe more complex techniques in the Methods section.*
☒	☐	A description of all covariates tested
☐	☒	A description of any assumptions or corrections, such as tests of normality and adjustment for multiple comparisons
☐	☒	A full description of the statistical parameters including central tendency (e.g. means) or other basic estimates (e.g. regression coefficient) AND variation (e.g. standard deviation) or associated estimates of uncertainty (e.g. confidence intervals)
☐	☒	For null hypothesis testing, the test statistic (e.g. *F*, *t*, *r*) with confidence intervals, effect sizes, degrees of freedom and *P* value noted<br>*Give P values as exact values whenever suitable.*
☒	☐	For Bayesian analysis, information on the choice of priors and Markov chain Monte Carlo settings
☒	☐	For hierarchical and complex designs, identification of the appropriate level for tests and full reporting of outcomes
☒	☐	Estimates of effect sizes (e.g. Cohen's *d*, Pearson's *r*), indicating how they were calculated

Our web collection on statistics for biologists contains articles on many of the points above.

Software and code

Policy information about availability of computer code

Data collection	For immunoblot acquisition: ImageStudioLite Odyssey software (v 5.2.5); For confocal image acquisition: Zeiss Zen blue (v 2.6). For qPCR acquisition: Biorad CFX Manager (v 3.1); For flow cytometry acquisition: BD CellQuest Pro (v 6)
Data analysis	ImageStudioLite Odyssey software (v 5.2.5) was used to analyze immunoblots. ImageJ (NIH) was used for all image analysis. FlowJo X was used for all flow cytometry analysis. Salmon (v 1.0.0) and DESeq (v 1.14.1) in RStudio were used for RNA-seq analysis.

For manuscripts utilizing custom algorithms or software that are central to the research but not yet described in published literature, software must be made available to editors and reviewers. We strongly encourage code deposition in a community repository (e.g. GitHub). See the Nature Portfolio guidelines for submitting code & software for further information.

Data

Policy information about availability of data

All manuscripts must include a data availability statement. This statement should provide the following information, where applicable:

- Accession codes, unique identifiers, or web links for publicly available datasets
- A description of any restrictions on data availability
- For clinical datasets or third party data, please ensure that the statement adheres to our policy

The RNA-seq data generated in this study have been deposited in the NIH GEO database under accession code GSE235265. The RNA-seq data (lytic time course) used in this study are available in the NIH GEO database under accession code GSE240008 and the proteomic data used in this study are available in the

ProteomeXchange Consortium PRIDE database under accession code PXD006317. The immunoblot and P-value data generated in this study are provided in the Source Data file. All figures were made using commercially available GraphPad, Adobe Illustrator, or Biorender.

Research involving human participants, their data, or biological material

Policy information about studies with human participants or human data. See also policy information about sex, gender (identity/presentation), and sexual orientation and race, ethnicity and racism.

Reporting on sex and gender	N/A
Reporting on race, ethnicity, or other socially relevant groupings	N/A
Population characteristics	N/A
Recruitment	N/A
Ethics oversight	N/A

Note that full information on the approval of the study protocol must also be provided in the manuscript.

Field-specific reporting

Please select the one below that is the best fit for your research. If you are not sure, read the appropriate sections before making your selection.

☒ Life sciences ☐ Behavioural & social sciences ☐ Ecological, evolutionary & environmental sciences

For a reference copy of the document with all sections, see nature.com/documents/nr-reporting-summary-flat.pdf

Life sciences study design

All studies must disclose on these points even when the disclosure is negative.

Sample size	All experiments were conducted in a minimum of two biological replicates, and most quantitative assays were performed in 3-4 biological replicates to enable accurate statistical measurements. Sample sizes for immunoblot and microscopy were based on guidelines in the peer-reviewed field.
Data exclusions	No data was excluded
Replication	All data represent biological replicates, as indicated in the figure legends and methods. Error bars represent standard deviation of at least three biological replicates unless otherwise stated.
Randomization	For every experiment, control samples were analyzed in parallel with the sample undergoing treatment/perturbation. Samples were randomly allocated to either control or treatment conditions and then labeled and treated with the same parameters (eg, cell growth conditions, lysis conditions, library preparation, etc.) except for the specific perturbation being tested (eg, KO of a given gene). Specific details for each experiment are described in the Methods.
Blinding	Investigators were not blinded during sample collection as the samples needed to be treated with specific lentiviruses and collected at specific time points. However, all experiments were performed in biological replicates across years of experiments.

Reporting for specific materials, systems and methods

We require information from authors about some types of materials, experimental systems and methods used in many studies. Here, indicate whether each material, system or method listed is relevant to your study. If you are not sure if a list item applies to your research, read the appropriate section before selecting a response.

Materials & experimental systems

n/a	Involved in the study
☐	☒ Antibodies
☐	☒ Eukaryotic cell lines
☒	☐ Palaeontology and archaeology
☒	☐ Animals and other organisms
☒	☐ Clinical data
☒	☐ Dual use research of concern
☒	☐ Plants

Methods

n/a	Involved in the study
☒	☐ ChIP-seq
☐	☒ Flow cytometry
☒	☐ MRI-based neuroimaging

Antibodies

Antibodies used

Primary antibodies: SFPQ (Bethyl A301-320A, Rb, 1:2000), SFPQ (Proteintech 15585-1-AP, Rb, 1:150 immunofluoresence), BMRF1 (OT14E2, gift from Dr. Jaap Middeldorp, Ms 1:1000 immunoblot and 1:500 immunofluoresence), BMRF1 (Santa Cruz sc-58121, Ms, 1:200 immunoblot and 1:500 immunofluoresence), BZLF1 (Santa Cruz sc-53904, Ms, 1:200 immunoblot and 1:500 immunofluoresence), p18 (Invitrogen PA1-73003, Gt, 1:500), EBNA2 (monoclonal antibody clone PE2, kindly provided by Dr. Jeffrey Cohen, Ms), ORF57 (Rockland 600-401-A94S, Rb, 1:1000 immunoblot and 1:200 immunofluoresence), H1.2 (GeneTex GTX122561, Rb, 1:1000), H1.4 (Cell Signaling D4J5Q, Rb, 1:1000), H3 (Cell Signaling D1H2, Rb, 1:1000), GAPDH (Cell Signaling D16H11, Rb, 1:1000), GAPDH (Millipore MAB374, Ms, 1:20,000), IFIT1 (Cell Signaling D2x9z, Rb, 1:500), IRF7 (Cell Signaling D2A1J, Rb, 1:1000), HA (Biolegend HA.11 clone 16B12, Ms, 1:200), HA (Cell Signaling C29F4, Rb, 1:1000 immunoblot and 10 uL for ChIP), V5 (Cell Signaling D3H8Q #13202, Rb, 1:1000), NONO (Proteintech 11058-1-AP, Rb, 1:1000), H1.2 (Proteintech 19649-1-AP, Rb, 2 ug for ChIP), normal IgG (Cell Signaling 2729S, Rb, 2 ug for ChIP), gp350 (BioXCell (72A1), Ms, 1:1000), V5 (Cell Signaling E9H8O, Ms, 1:100), c-MYC (Cell Signaling D84C12, Rb, 1:1000)

Secondary antibodies: Anti-rabbit IgG, HRP-linked Antibody (Cell Signaling 7074V, 1:4000), Anti-mouse IgG, HRP-linked Antibody (Cell Signaling 7076V, 1:4000), HRP anti-Goat (Thermo Fisher #A24452, 1:5000), IRDye® 680RD Goat anti-Rabbit IgG (H + L) (Li-COR Inc 926-68071, 1:10,000), IRDye® 800CW Goat anti-Mouse IgG (H + L) (LI-COR Inc 926-32210, 1:10,000), Goat anti-Rabbit IgG (H+L) Cross-Adsorbed Secondary Antibody, Alexa Fluor 488 (Invitrogen, 1:500), Goat anti-Mouse IgG (H+L) Secondary Antibody, Alexa Fluor 488 (Invitrogen, 1:500), Goat anti-Rabbit IgG (H+L) Cross-Adsorbed Secondary Antibody, Alexa Fluor 568 (Invitrogen, 1:500), Goat anti-Mouse IgG (H+L) Secondary Antibody, Alexa Fluor 568 (Invitrogen, 1:500)

Validation

All commercially available antibodies have been validated by the companies that sell them mentioned above and detailed data about the validation (immunoblot, immunofluoresence, KO) can be found on the company's websites and in the papers that they cite on their websites. In addition, we validated the SFPQ, NONO, H1.2, H1.4, and BLZF1 antibodies via immunoblot in a KO background. Furthermore, all of the antibodies that were gifts have been validated in EBV positive versus negative cell lines.

Eukaryotic cell lines

Policy information about cell lines and Sex and Gender in Research

Cell line source(s)

All B-cell lines were Cas9+. With the exception of the (EBV-) MUTU I and Ramos cell lines, all Cas9+ cell lines were previously generated. The B-cell lines used in this study are as follows with the sources indicated: P3HR-1-ZHT/RHT Cas9+ (original cell line gift from Drs. Elliott Kieff and Eric Johannsen), MUTU I (EBV+) (original cell line gift from Dr. Jeff Sample), MUTU I (EBV-) Cas9+ (original cell line gift from Dr. Bill Sugden), Akata EBV+ Cas9+ (original cell line gift from Dr. Elliott Kieff), Daudi EBV+ Cas9+ (original cell line from ATCC, CCL-213), Ramos Cas9+ (original cell line gift from Dr. Karl Munger), and JSC-1 Cas9+ (gift from Dr. Kenneth Kaye). The other cell lines used in the study are from the following sources: C666.1 (gift from Dr. Elliott Kieff), SNU-719 (gift from Dr. Adam Bass), YCCE1L (gift from Dr. Elliott Kieff), and hTERT-immortalized NOK (gift from Dr. Elliott Kieff), 293T (ATCC).

Authentication

Cell lines were not authenticated beyond confirming that the EBV+ cells were EBV+, and the EBV- cell lines were EBV-. Cell lines expressing new constructs (KO or OE) were validated via WB.

Mycoplasma contamination

Cell lines tested negative for mycoplasma.

Commonly misidentified lines
(See ICLAC register)

No commonly misidentified cell lines were used in this study.

Plants

Seed stocks: N/A

Novel plant genotypes: N/A

Authentication: N/A

Flow Cytometry

Plots

Confirm that:

☒ The axis labels state the marker and fluorochrome used (e.g. CD4-FITC).

☒ The axis scales are clearly visible. Include numbers along axes only for bottom left plot of group (a 'group' is an analysis of identical markers).

☒ All plots are contour plots with outliers or pseudocolor plots.

☒ A numerical value for number of cells or percentage (with statistics) is provided.

Methodology

Sample preparation: Sample preparation is detailed in the methods section.

Instrument: BD FACSCalibur

Software: FlowJo X

Cell population abundance: N/A, no sorting was done

Gating strategy: All gating is shown in the supplementary material.

☒ Tick this box to confirm that a figure exemplifying the gating strategy is provided in the Supplementary Information.
